# Precision-engineered PROTACs minimize off-tissue effects in cancer therapy

**DOI:** 10.3389/fmolb.2024.1505255

**Published:** 2024-11-22

**Authors:** Jianghua Shi, Luo Wang, Xuanwei Zeng, Chengzhi Xie, Zhaowei Meng, Anahit Campbell, Lulu Wang, Heli Fan, Huabing Sun

**Affiliations:** ^1^ National Engineering and Technology Research Center of Chirality Pharmaceutical, Lunan Pharmaceutical Group Co., Ltd., Linyi, China; ^2^ State Key Laboratory of Experimental Hematology, Tianjin Key Laboratory on Technologies Enabling Development of Clinical Therapeutics and Diagnostics, The School of Pharmacy, Tianjin Medical University, Tianjin, China; ^3^ Department of Nuclear Medicine, Tianjin Medical University General Hospital, Tianjin, China; ^4^ Department of Chemistry and Biochemistry, University of Wisconsin-Milwaukee, Milwaukee, WI, United States

**Keywords:** PROTACs, off-tissue effects, precision-engineered, targeted protein degradation, PRO-PROTAC, redox-inducible

## Abstract

Proteolysis-targeting chimeras (PROTACs) offer a groundbreaking approach to selectively degrade disease-related proteins by utilizing the ubiquitin-proteasome system. While this strategy shows great potential in preclinical and clinical settings, off-tissue effects remain a major challenge, leading to toxicity in healthy tissues. This review explores recent advancements aimed at improving PROTAC specificity, including tumor-specific ligand-directed PROTACs, pro-PROTACs activated in tumor environments, and E3 ligase overexpression strategies. Innovations such as PEGylation and nanotechnology also play a role in optimizing PROTAC efficacy. These developments hold promise for safer, more effective cancer therapies, though challenges remain for clinical translation.

## 1 Introduction

Proteolysis-targeting chimeras (PROTACs) represent a breakthrough in targeted protein degradation, leveraging the ubiquitin-proteasome system (UPS) to eliminate disease-causing proteins, many of which are considered “undruggable” by conventional therapies (Chirnomas et al., 2023; Diehl and Ciulli, 2022). Composed of a target protein ligand, an E3 ligase ligand, and a linker, PROTACs promote the ubiquitination and degradation of disease-related proteins by bringing them into proximity with an E3 ligase (Yan et al., 2024; Li et al., 2022).

**FIGURE 1:**
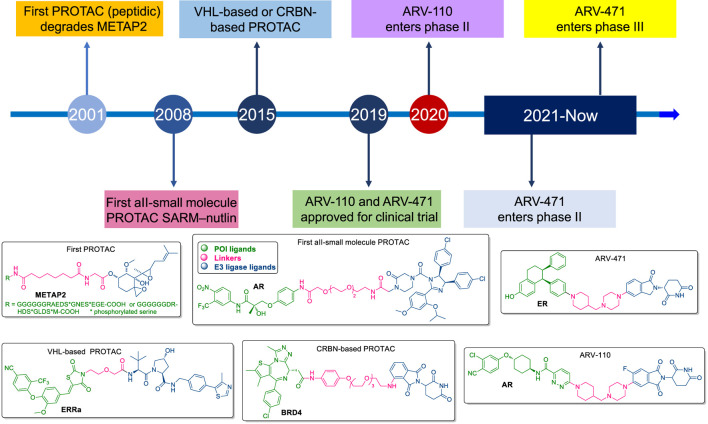
Milestones in the development of PROTACs.

Introduced in the early 2000s by Craig Crews, the first generation of PROTACs was peptidic, targeting proteins like METAP2 (Sakamoto et al., 2001) (Figure 1). While promising, these early PROTACs were large, lacked cell permeability, and were rapidly degraded by proteases, limiting their bioavailability. The introduction of small-molecule PROTACs, such as SARM-nutlin in 2008, addressed many of these limitations by improving pharmacokinetics, cell permeability, and overall efficacy, broadening the scope of therapeutic applications to diseases like cancer (Schneekloth et al., 2008). The development of VHL-based and CRBN-based PROTACs marked significant milestones in this technology. The first VHL-based PROTAC, targeting the androgen receptor (AR) in prostate cancer, was developed in 2012, while the first CRBN-based PROTAC, targeting BRD4, followed in 2015 (Bondeson et al., 2015; Lu et al., 2015). These innovations demonstrated the ability of PROTACs to selectively degrade target proteins, setting the stage for the rapid advancement of the field. ARV-110 and ARV-471 are notable examples of PROTACs that have progressed to clinical trials, focusing on targeting the androgen receptor and estrogen receptor, respectively (Liu Z. et al., 2022) (Figure 1). These drugs hold the potential to overcome resistance mechanisms seen in traditional therapies, marking a significant step forward in cancer treatment and expanding the horizon for drugging previously “undruggable” proteins.

PROTACs have shown considerable promise in both preclinical and clinical settings (Békés et al., 2022; Hu and Crews, 2022; Shi et al., 2024). However, challenges remain, and a key concern is off-tissue degradation, where PROTACs may induce unwanted protein degradation in non-target tissues, leading to toxicity (Wang et al., 2024b; Zhang et al., 2024; Chen et al., 2023; Li et al., 2024). This risk is heightened by the ubiquitous expression of E3 ligases, which can ubiquitinate target proteins in both diseased and healthy tissues (Kannt and Dikic, 2021; Yu et al., 2024; Jiang et al., 2023). For instance, ARV-110, developed for prostate cancer, has shown efficacy but underscored the need for greater precision to avoid degradation in normal tissues and reduce side effects (Chen et al., 2024; Huang et al., 2022). Additionally, neutropenia was observed as a side effect during the clinical trial of CFT7455 (Li K. et al., 2022).

**FIGURE 2:**
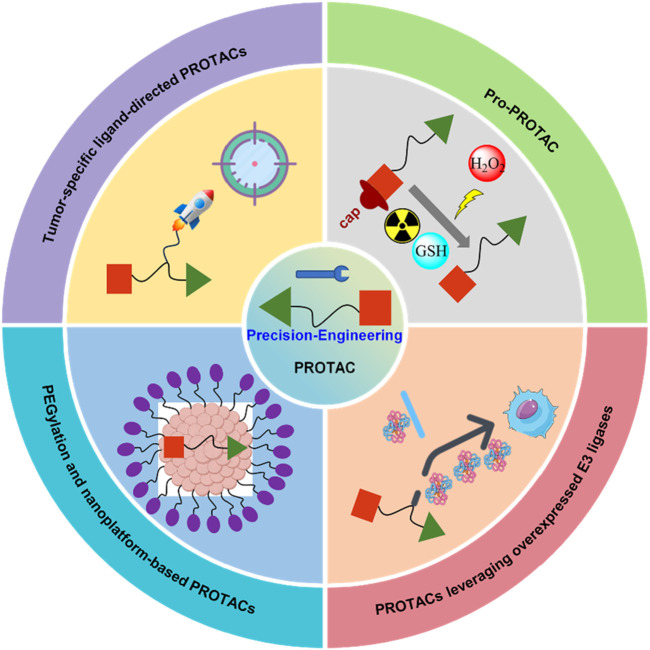
Strategies to reduce off-tissue toxicity of PROTACs through precision-engineering.

To address these challenges, several strategies are being developed, including tumor-specific ligand-directed PROTACs, pro-PROTACs that are selectively activated in target tissues, and exploiting overexpressed E3 ligases in certain cancers (Yim et al., 2024) (Figure 2). Additionally, approaches like PEGylation and nanotechnology are being employed to improve pharmacokinetics, reduce non-specific binding, and enhance the selectivity of protein degradation, thereby expanding the therapeutic window (Li D. et al., 2022; Zhang et al., 2023; Zhang et al., 2022) (Figure 2). These efforts reflect the evolving landscape of PROTAC technology as researchers strive to balance potency with safety through chemically engineering, aiming for more precise and effective cancer therapies.

## 2 Precision-engineered PROTACs

### 2.1 Tumor-specific ligand-directed PROTACs

**FIGURE 3:**
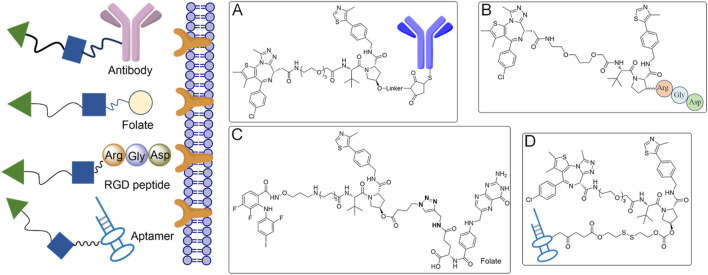
Tumor-specific ligand-directed PROTACs by antibody, folate, RGD peptide and aptamer. **(A)** The PROTACs achieve targeted delivery to cancer cells via antibody-mediated recognition. **(B)** Folate receptors on cancer cells are exploited by the PROTACs for selective targeting. **(C)** The PROTACs utilize RGD peptides to specifically bind to integrins on cancer cells. **(D)** Aptamers are employed by the PROTACs for precise cancer cell targeting.

To minimize off-target toxicity, a key strategy is the chemical conjugation of tumor-specific targeting ligands, which direct PROTACs to specific tumors, concentrating therapeutic effects while reducing adverse reactions elsewhere (Zhao et al., 2020) (Figure 3). Antibody-conjugated PROTACs (Ab-PROTACs), for example, target overexpressed cell surface antigens in cancer cells (Chang et al., 2023; Guo et al., 2024). After binding, the PROTAC moiety is internalized and degrades intracellular proteins (Figure 3A). HER2-targeted Ab-PROTACs selectively degrade proteins in HER2-positive cancer cells, sparing healthy tissues in cancer therapy (Hu et al., 2022; Tsuchikama et al., 2024). Despite their potential, the large size of antibodies poses challenges like reduced stability, immunogenicity, and difficulty in internalization, all of which must be addressed to enhance their therapeutic viability (Elbakri et al., 2010). Beyond Ab-PROTACs, small molecule-based PROTACs conjugated with tumor-specific ligands, such as folate, show promising results (Figure 3B). Folate conjugation, for instance, selectively targets folate receptor-overexpressing cancer cells, allowing localized protein degradation and minimizing systemic toxicity (Liu et al., 2021). RGD (Arg-Gly-Asp) peptides are known to bind α(v)β(3) integrin, which is overexpressed in many tumors and play a crucial role in tumor angiogenesis and metastasis (Figure 3C). Similarly, RGD peptide-conjugated PROTACs target integrin-expressing tumors, selectively degrading proteins like BCL-xL in these cancer cells (Zanella et al., 2019; Danhier et al., 2012).

Aptamer-based PROTACs offer another innovative approach (Liu et al., 2023a) (Figure 3D). Aptamers, single-stranded nucleic acids with high specificity for targets, can guide PROTACs to specific tissues. An aptamer-conjugated PROTAC designed to degrade HER2 protein has been shown to selectively target HER2-positive breast cancer cells, enhancing treatment precision and reducing off-target effects (He et al., 2021; Li et al., 2023). Beyond cancer, aptamer-based PROTACs could be further explored in other diseases where tumor-specific protein degradation is critical.

These advancements in tumor-specific ligand-directed PROTACs could make PROTAC therapies more precise and safer, paving the way for broader applications in personalized medicine. Further optimization of bio-stability, conjugation techniques, and POI degradation efficiency will be crucial to fully realizing their potential in clinical settings (Joubert et al., 2017).

### 2.2 Pro-PROTAC approaches

**FIGURE 4:**
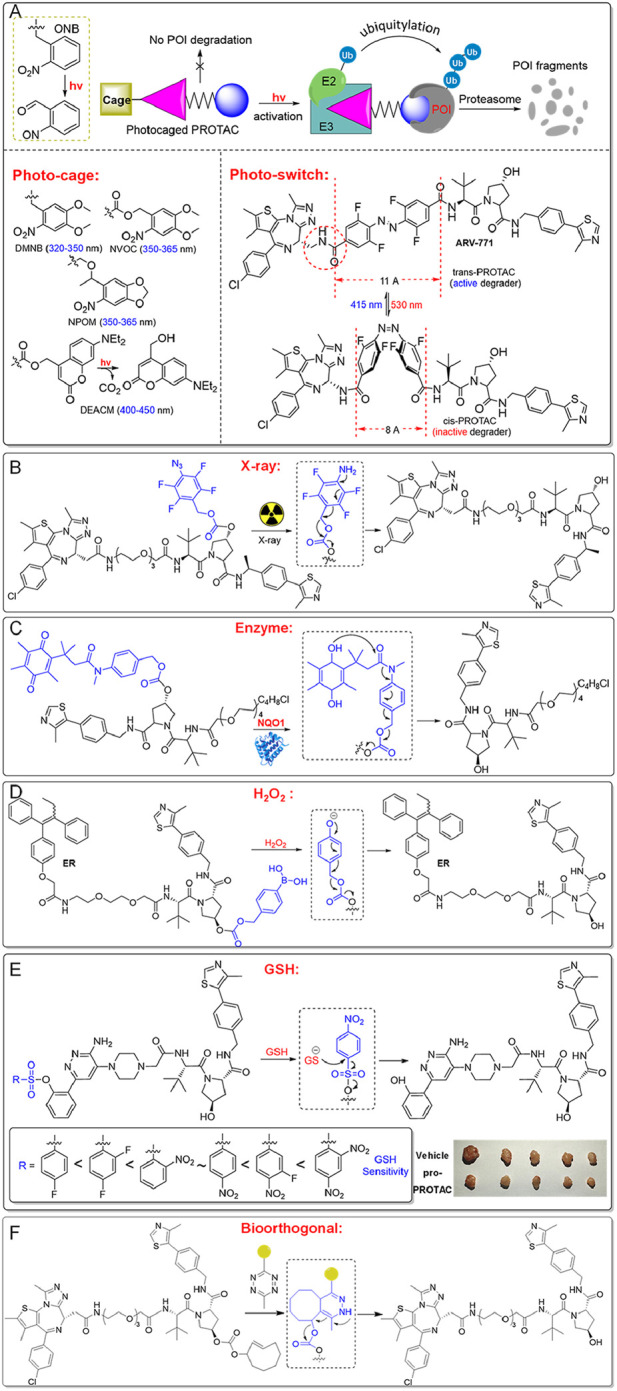
Pro-PROTACs allow for selective activation in cancer cells. **(A)** Pro-PROTACs can be activated specifically through photolysis. **(B)** Pro-PROTACs can be activated by exposure to X-ray irradiation. **(C)** Pro-PROTACs can undergo activation via targeted enzymatic reactions. **(D)** Pro-PROTACs can be activated by elevated hydrogen peroxide in cancer cells. **(E)** Pro-PROTACs can be activated by intracellular GSH, exploiting its high levels in cancer cells. **(F)** Pro-PROTACs can be triggered through bioorthogonal chemical reactions.

Pro-PROTAC approach offers a promising strategy to minimize systemic toxicity by ensuring activation primarily within the target tissue (Chen et al., 2023) (Figure 4). These inactive PROTAC precursors remain inert until selectively activated by specific environmental triggers, such as light, X-ray, or the unique enzymatic (e.g., NQO1) and chemical (e.g., redox state) conditions in tumor cells (An et al., 2023; Saxon and Peng, 2022). This is especially advantageous in cancer therapy.

Photo-PROTACs provide a straightforward method to achieve spatial and temporal control over protein degradation. Photo-cage PROTACs typically feature a photolabile “cage” that blocks the active site of the PROTAC (Xue et al., 2019) (Figure 4A). Upon exposure to a specific wavelength of light, the photocage is removed, releasing the active PROTAC to degrade target proteins. Common photolabile groups include Ortho-Nitrobenzyl (ONB, 300–365 nm), Nitroveratryloxycarbonyl (NVOC, 350–365 nm), 6-Nitropiperonyloxymethyl (NPOM, 350–365 nm), 4,5-Dimethoxy-2-nitrobenzyl (DMNB, 320–350 nm), and Diethylaminocoumarin (DEACM, 400–450 nm) (Klán et al., 2013; Liu et al., 2020; Naro et al., 2020). These photo-cage PROTACs remain inactive until illuminated, enabling precise control over when and where protein degradation occurs.

Additionally, azobenzene-based photo-switch PROTACs (Azo-PROTACs) offer reversible control of protein degradation (Jin et al., 2020; Pfaff et al., 2019). These PROTACs incorporate azobenzene moieties between ligands targeting the E3 ligase and the protein of interest (Figure 4A). Light induces conformational changes in the azobenzene, switching the PROTAC between active and inactive states. For instance, the trans-isomer (415 nm) enables effective protein degradation, while the cis-isomer (530 nm) is inactive due to a shorter linker distance that prevents the necessary protein-protein interactions. Photo-switch PROTACs have shown efficacy in degrading target proteins like BCR-ABL in leukemia cells, underscoring their potential for precise therapeutic applications.

Using light as a trigger for PROTACs allows precise control over the timing and location of protein degradation, but it faces limitations such as poor tissue penetration of UV/visible light and potential phototoxicity (Min et al., 2015). Near-infrared (NIR) and two-photon techniques offer alternatives, but their efficiency remains too low for practical clinical use (Shaw et al., 2022). X-ray radiation-responsive PROTACs, on the other hand, take advantage of X-ray’s deep tissue penetration (Geng et al., 2021). These PROTACs typically contain radiation-cleavable groups, such as phenyl azide, which block the interaction between the PROTAC and its target or the E3 ligase (Figure 4B). Upon exposure to X-ray during radiotherapy, the phenyl azide group is reduced, releasing the active PROTAC. This approach enhances specificity and shows synergistic antitumor effects, as demonstrated in MCF-7 xenograft models (Yang et al., 2023).

Pro-PROTACs, designed to respond to the unique enzymatic or chemical environment of tumor cells, are another promising strategy (Liang et al., 2022; Fan et al., 2023) (Figure 4). For example, NQO1, an enzyme overexpressed in certain cancers, can activate NQO1-PROTACs by cleaving their caging groups (Figure 4C). This releases the active PROTAC, enabling it to bind target proteins like HDAC6 and recruit an E3 ligase for degradation (Jia et al., 2023b). This strategy ensures that the PROTAC remains inactive in normal cells, reducing systemic toxicity and off-target effects, offering high selectivity for cancer cells.

Redox-sensitive PROTACs offer a sophisticated and selective strategy for cancer treatment by exploiting the distinct redox imbalance in tumor cells (Figure 4D). Cancer cells often exhibit elevated levels of hydrogen peroxide (H_2_O_2_) and glutathione (GSH), which are essential for maintaining their altered redox homeostasis (Yu et al., 2023a; Ali et al., 2024; Wang et al., 2017; Chen et al., 2018; Xue et al., 2023; Jia et al., 2023a). This disparity between cancerous and normal tissues provides a unique opportunity for targeted therapeutic intervention (Saxon et al., 2024; Cao et al., 2023; Sun et al., 2024). Typically, H_2_O_2_-sensitive PROTACs are designed with H_2_O_2_-responsive linkers or protective groups, such as aryboronates/boronic acids (converted into phenol group), or phenyl selenide (converted into benzeneselenenic acid), which act as cage units to mask the active PROTAC moiety (Yu et al., 2023b; Yu et al., 2022). Upon reacting with elevated levels of hydrogen peroxide (H_2_O_2_) in tumor cells, these groups are de-caged, releasing active PROTACs. For instance, H_2_O_2_-inducible PROTAC precursors release active PROTACs in cancer cells, such as A549 and H1299, leading to selective degradation of BRD4 or the estrogen receptor, inducing cytotoxicity in cancer cells while sparing H_2_O_2_-deficient normal cells, such as WI38. This mechanism enhances therapeutic precision by minimizing off-target effects in healthy tissues.

Encouraging results have also been observed with GSH-responsive PROTACs, which utilize benzenesulfonyl cage groups to block PROTAC activity (Xue et al., 2022; Chai et al., 2024). These groups are activated in the glutathione (GSH)-rich environment of cancer cells, particularly in lung cancer, restoring PROTAC functionality (Figure 4E). The reactivity of these precursors can be fine-tuned by introducing electron-withdrawing substituents, such as -F or -NO_2_, on the benzenesulfonyl unit to increase sensitivity to GSH (Fan et al., 2023; Sun et al., 2024; Zhou et al., 2023). For example, a GSH-inducible SMARCA2/4-targeting PROTAC demonstrates selective degradation of SMARCA2/4 in cancer cells, promoting DNA damage and apoptosis while inhibiting tumor growth in xenograft models without adverse effects on normal cells (Ji et al., 2024). This redox-inducible strategy marks a significant advancement in PROTAC design, providing a versatile and effective approach for improving therapeutic precision in cancer treatment (Figure 4E). By enhancing selectivity and reducing off-target toxicity, redox-sensitive PROTACs represent a promising avenue for the development of safer, more effective cancer therapies.

Bioorthogonal pro-PROTACs are another cutting-edge method in targeted cancer therapy, enabling the precise activation of PROTACs within cancer cells via bioorthogonal chemical reactions with exogenous chemical agents (Bi et al., 2023; Huang et al., 2023) (Figure 4F). For instance, inactive pro-PROTACs like TCO-ARV-771 and TCO-DT2216, which are conjugated with a bioorthogonal trans-cyclooctene (TCO) group, can be selectively activated by tetrazine (Tz)-modified RGD peptide, c(RGDyK)-Tz, in cancer cells expressing the integrin αvβ3 biomarker. Once activated, the prodrugs release active PROTACs, promoting targeted protein degradation within cancer cells while minimizing off-target effects on healthy cells (Chang et al., 2023). This approach allows for spatially and temporally controlled PROTAC activation, thereby improving therapeutic specificity and reducing unintended toxicity. These advancements pave the way for the development of more selective and efficient cancer treatments, offering significant potential for future biomedical applications.

### 2.3 PROTACs targeting overexpressed E3 ligases

**FIGURE 5:**
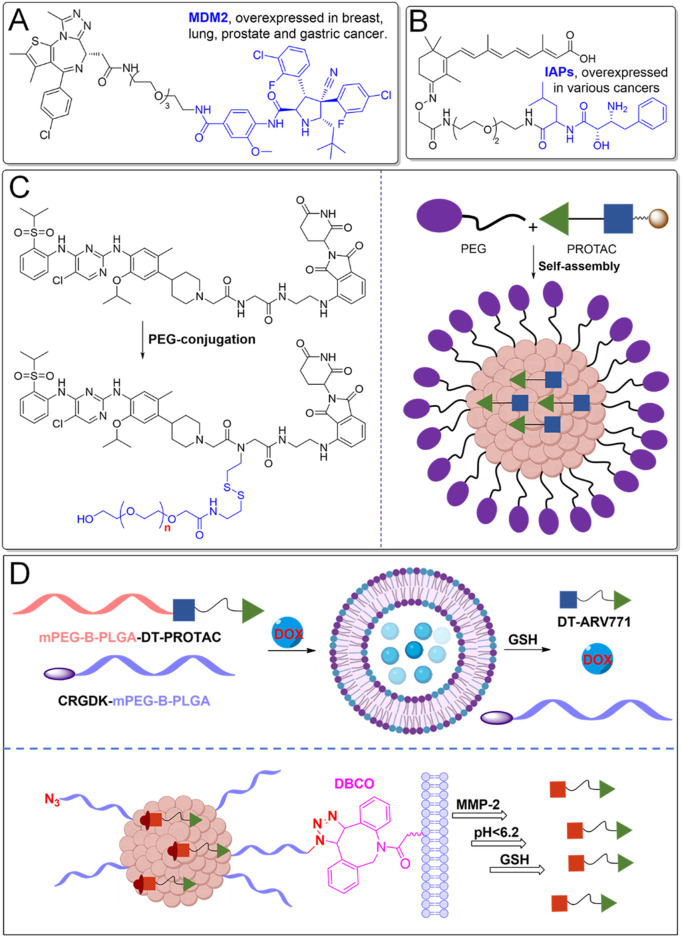
PROTACs leveraging overexpressed E3 ligases and nanotechnology. **(A)** PROTACs leverage overexpressed E3 ligases, such as MDM2, in certain cancers to enhance their targeted protein degradation. **(B)** PROTACs utilize overexpressed IAPs in specific cancers for therapeutic purposes. **(C)** PEGylation significantly enhances the pharmacokinetic properties of PROTACs. **(D)** Nanotechnology facilitates better delivery and bioavailability.

E3 ubiquitin ligases are critical components of the ubiquitin-proteasome system (UPS), responsible for ubiquitination and subsequent degradation of target proteins. PROTACs that exploit overexpressed E3 ubiquitin ligases in cancer cells have emerged as a novel therapeutic strategy with improved selectivity (Figure 5). Overexpression of specific E3 ligases, such as MDM2 and IAPs, makes them attractive targets for selective degradation of oncogenic proteins through PROTAC technology (Liu et al., 2023b).

MDM2-targeting PROTACs exploit the overexpression of the MDM2 ligase, common in cancers such as sarcomas and gliomas, which typically degrade the tumor suppressor protein p53 (Shangary and Wang, 2008) (Figure 5A). In these cancers, p53’s tumor-suppressive function is impaired, contributing to unchecked proliferation (Traweek et al., 2022). MDM2-based PROTACs redirect MDM2 to degrade oncogenic proteins like BRD4, while simultaneously restoring p53’s activity. This dual action is particularly beneficial in cancers with intact but functionally inactive p53, offering a promising route for therapeutic intervention (Hines et al., 2019).

IAPs, including cIAP1, cIAP2, and XIAP, also serve as E3 ligases and regulators of apoptosis (Liu X. et al., 2022) (Figure 5B). Overexpressed in many cancers, they contribute to resistance against apoptosis. IAP-based PROTACs, designed to target oncogenic proteins such as BCL-XL, provide a more selective alternative to VHL- or CRBN-based PROTACs (Zhang et al., 2020). This approach reduces the risk of platelet toxicity, a common side effect with other ligases, making IAP-based PROTACs a promising option for targeting proteins like BCL-XL in cancer therapies, while minimizing toxicity (Ma et al., 2021). It should be noted that only a limited number of E3 ligases have been effectively utilized in PROTAC designs, restricting their applicability to some cancers, as these ligases are not universally overexpressed across all cancer types. Expanding the pool of E3 ligases, particularly those with tumor-specific expression, could significantly enhance the precision and therapeutic scope of PROTACs. Moreover, advanced delivery systems, such as nanoparticle carriers, may further improve the tumor-targeting efficiency of PROTACs by increasing their selective accumulation in cancerous tissues, thus reducing systemic toxicity and enhancing overall therapeutic efficacy.

### 2.4 PEGylation and nanotechnology-based PROTACs

Optimizing the pharmacokinetics of PROTACs through PEGylation and nanotechnology can effectively reduce off-target accumulation, minimizing unintended effects (Cecchini et al., 2022) (Figure 5C). These modifications enhance hydrophilicity, decrease non-specific binding, and extend circulation time, allowing for more selective tissue uptake (Chen et al., 2022). PEGylation improves the solubility and stability of PROTACs by enabling them to self-assemble into micelles, which remain stable in circulation and release the active PROTAC in response to elevated glutathione (GSH) levels in tumor environments. Preclinical studies have shown superior bioavailability (84.8%) compared to unmodified versions, significantly reducing tumor size in xenograft models, indicating strong potential for cancer therapy (Wang et al., 2024a).

Nano-PROTACs further enhance tumor specificity by leveraging nanotechnology (Zhang et al., 2023; Song et al., 2024). For example, SPNpro, a self-assembling nano-PROTAC, incorporates a semiconductor polymer core that generates singlet oxygen upon light activation, killing tumor cells (Figure 5C). A cancer biomarker-responsive peptide cleaves SPNpro, releasing active PROTACs to degrade immunosuppressive targets such as IDO, reversing immune suppression and promoting antitumor immunity (Zhang et al., 2021). This strategy also mitigates issues like the “hook effect” by enabling dose-dependent protein degradation. In preclinical models, Nano-PROTACs demonstrated a 95% degradation rate with long-lasting potency and significant tumor inhibition. Additionally, phototherapeutic nano-PROTACs, like those targeting COX-1/2 and activated by tumor-overexpressed cathepsin B, enhance tumor-specific protein degradation, reprogram the tumor microenvironment (TME), and stimulate immune responses, further suppressing tumor growth (Gao et al., 2024).

To further improve selectivity in cancer therapy, advanced nano-PROTAC combination therapy approaches and multiple stimuli-responsive nano-PROTAC systems have been developed (Figure 5D). Nano-PROTAC combination therapy enables the co-delivery of a PROTAC degrader alongside conventional small-molecule drugs, allowing for synergistic effects (Zou et al., 2024). Self-assembling pro-PROTAC nanoparticles are engineered to specifically target tumor cells, where they can be activated to release the free PROTACs, facilitating precise protein degradation. Additionally, multiple stimuli-responsive nano-PROTAC systems allow for enhanced precision in protein degradation. For example, an azide-modified nano-PROTAC utilizes bioorthogonal click chemistry to amplify PROTAC delivery selectively to tumor tissues (Gao et al., 2022). These nano-PROTACs can sequentially respond to extracellular matrix metalloproteinase-2 (MMP-2), acidic intracellular environments (pH < 6.2), and the reductive tumor microenvironment (GSH) to release the active PROTACs. This targeted release facilitates tumor-specific BRD4 degradation, offering a highly selective and efficient approach to PROTAC-based cancer therapy.

These findings highlight the potential of PEGylation and nano-PROTACs in overcoming the traditional limitations of PROTACs, such as poor bioavailability and solubility, while enhancing therapeutic efficacy. However, introducing PEGylation and nanoplatforms raises concerns about biocompatibility, immune response, and safety, which may complicate their clinical application (Ernst et al., 2021). Balancing efficacy with these potential risks is crucial for the future of these innovative therapies.

## 3 Conclusion

PROTACs offer a revolutionary approach to targeted protein degradation, showing great promise in both preclinical and clinical studies. However, challenges such as off-tissue effects and systemic toxicity remain obstacles to clinical success. Recent strategies, such as tumor-specific ligand-directed PROTACs, E3 ligase-specific designs, pro-PROTACs, and nanotechnology are helping to mitigate these issues by enhancing selectivity and reducing unwanted degradation. Despite encouraging progress, more advancements are necessary, particularly in developing biocompatible linkers, novel ligands, and exploring new E3 ligases to improve specificity and reduce toxicity, essential for clinical translation.
